# Resting-state functional brain alterations in functional dyspepsia: Protocol for a systematic review and voxel-based meta-analysis

**DOI:** 10.1097/MD.0000000000023292

**Published:** 2020-11-25

**Authors:** Ruirui Sun, Jie Zhou, Yuzhu Qu, Jun Zhou, Guixing Xu, Shirui Cheng

**Affiliations:** a The Acupuncture and Tuina School/The 3rd Teaching Hospital, Chengdu University of Traditional Chinese Medicine, Chengdu; b The Third Clinical Medical College, Zhejiang Chinese Medical University, Zhejiang, China.

**Keywords:** functional brain alterations, functional dyspepsia, neuroimaging, signed differential mapping

## Abstract

**Background:**

Functional dyspepsia (FD) is one of the most common functional gastrointestinal disorders (FGIDs) and significantly influences patients’ quality of life. Many studies have found that patients with FD show significant functional abnormalities in multiple brain regions. However, these functional cerebral abnormalities are not fully consistent. This protocol aims to qualitatively and quantitatively assess and synthesize the functional cerebral abnormalities found in FD.

**Methods:**

A systematic search will be conducted in 4 electronic databases (Medline, Web of Science, EMBASE, and the Cochrane Library) from inception to June 30, 2019, with the language restricted to English. Study selection will follow the Preferred Reporting Items for Systematic Reviews and Meta-Analyses (PRISMA) guidelines. Quality assessment will be performed with a custom 11-point checklist. The functional changes in brain regions and the correlations between these altered brain regions and clinical variables in patients with FD will be evaluated through qualitative review. If data are available, an Anisotropic Effect Size version of Signed Differential Mapping (AES-SDM) will be used to synthesize the brain functional alterations and clinical variables in patients with FD.

**Results:**

This review and meta-analysis will qualitatively and quantitatively assess and synthesize functional cerebral abnormalities consistently found in FD.

**Conclusion:**

This may assist in mapping functional brain abnormalities to characterize imaging-based neural markers of FD and improve our knowledge of the pathogenesis of FD.

**PROSPERO registration number:**

CRD42019134983 (https://www.crd.york.ac.uk/prospero/)

## 1 Introduction

Functional dyspepsia (FD) is one of the most common functional gastrointestinal disorders (FGIDs) with a high prevalence ranging from 5% to 25% globally.^[1–3]^ FD is defined as a chronic disease with the typical symptoms of postprandial fullness, early satiety, epigastric pain, and epigastric burning, in the absence of any organic, systemic, or metabolic disease according to the Rome IV criteria.^[4,5]^ FD is closely related to anxiety, depression, and reduced quality of life (QOL),^[6–8]^ and results in heavy economic burden to families and society.^[9,10]^

Recently, the theory of brain–gut interaction has been proposed to help better understand the pathogenesis of FD.^[11–13]^ The mechanism of FD is not only significantly involved in peripheral gut dysfunction but also functional brain abnormalities.^[14,15]^ Thus, the wide application of functional neuroimaging techniques can greatly enhance our understanding of the pathological role that functional brain changes have on FD. With positron emission tomography (PET), functional magnetic resonance imaging (fMRI), and single photon emission computed tomography (SPECT), investigators have found that patients with FD show significant functional abnormalities in multiple brain regions/cerebral networks, including but not limited, to the periaqueductal gray,^[16,17]^ dorsolateral prefrontal cortex,^[17]^ cingulate cortex,^[16–19]^ insula,^[17–19]^ thalamus,^[16,18,19]^ cerebellum, and default mode network.^[18,19]^ However, the results of these functional neuroimaging studies are heterogeneous. Methodological limitations such as scanning method, sample size, and data analysis might contribute to these inconsistent results. For example, patients with FD show a lower glycometabolism in the cingulate gyrus than healthy subjects (HS),^[20]^ while this region has been associated with higher glycometabolism in another study.^[21]^

A recent systematic review in 2016 integrated the abnormal central changes in FD and found heterogeneous altered brain regions.^[22]^ This protocol will mainly perform a meta-analysis to evaluate the consistency of variable results in resting-state neuroimaging studies and to explore the reason for this potential heterogeneity. Anisotropic effect size version of signed differential mapping (AES-SDM) is a method available to perform a meta-analysis of neuroimaging data. AES-SDM combines peak coordinates and statistical parametric maps and uses standard effect size, enabling an exhaustive inclusion and accurate estimations of studies.^[23–25]^ AES-SDM has a higher sensitivity, overlap, and good control of false positives, the imprecision of which has been demonstrated to be lower than other coordinated-based methods; this increases the reliability and validity of the neuroimaging results.^[24]^

Accordingly, this protocol aims to: update the systematic review from 2016^[22]^ and synthesize the current neuroimaging studies that investigated abnormal central changes in FD patients; compare regions most consistently and significantly distinguished in FD compared with HS by AES-SDM. The results may contribute to mapping functional brain characters of FD.

## 2 Methods and design

This protocol follows the Preferred Reporting Items for Systematic review and Meta-Analysis Protocols (PRISMA-P) statement.^[26]^ The protocol has been registered in the PROSPERO International Prospective Register of Systematic Reviews (http://www.crd.york.ac.uk/PROSPERO), registration number: CRD42019134983.

### 2.1 Search strategy

A comprehensive and exhaustive search will be conducted in Medline, Web of Science, EMBASE, and the Cochrane Library from database inception to June 30, 2019. The search strategy has been developed using Medical Subject Headings (MeSH); the Ovid Medline search strategy is shown in the Supplementary File. This search strategy will be modified to be suitable for other electronic databases. Relevant references cited in the selected studies will be carefully reviewed for potentially suitable studies. Any studies not identified by the computerized literature search will be checked manually. Conference proceedings and unpublished literature will also be checked and clearly documented for subsequent verification.

### 2.2 Eligibility criteria

Eligibility criteria for this review are presented with the following items: population, intervention/exposure, comparator, outcome, and study characteristics.

#### 2.2.1 Population

Participants will be limited to definite untreated patients with FD and age-matched HS. The age of participants will be restricted from 18 to 65 years.

#### 2.2.2 Exposure

Studies must include an FD sample, which is diagnosed based on the Rome criteria. Studies that involved participants who meet the criteria for other clinical diagnosed diseases will be excluded.

#### 2.2.3 Comparator

Inclusion of an age-matched HS group never diagnosed with FD is required for studies to be included in this review. Included studies must compare patients with FD with HS.

#### 2.2.4 Outcomes

The primary outcomes of the included studies are resting-state functional alterations in the brain regions of patients with FD. The cerebral function variables include blood-oxygen-level-dependent signal or cerebral blood flow (fMRI), and brain molecular metabolism (PET, SPECT). Included studies must perform whole-brain analysis, and the results should be presented in the Montreal Neurological Institute (MNI) or Talairach coordinates (*x*, *y*, *z*). The secondary outcomes of the included studies include the Nepean Dyspepsia Index (NDI), Self-Rating Anxiety Scale (SAS), and Self-Rating Depression Scale (SDS).

#### 2.2.5 Study characteristics

Original peer-reviewed functional neuroimaging studies published prior to June 30, 2019 will be included. The included studies in this review must be available in English. Case reports, qualitative studies, reviews or meta-analyses, and information in books or letters will be excluded. Studies performing structural brain analysis rather than functional brain analysis, using region of interest (ROI) approaches and/or various statistical thresholds in different brain regions, employing small volume corrections (SVC) in preselected ROIs will be excluded.

### 2.3 Date selection

**Figure 1:**
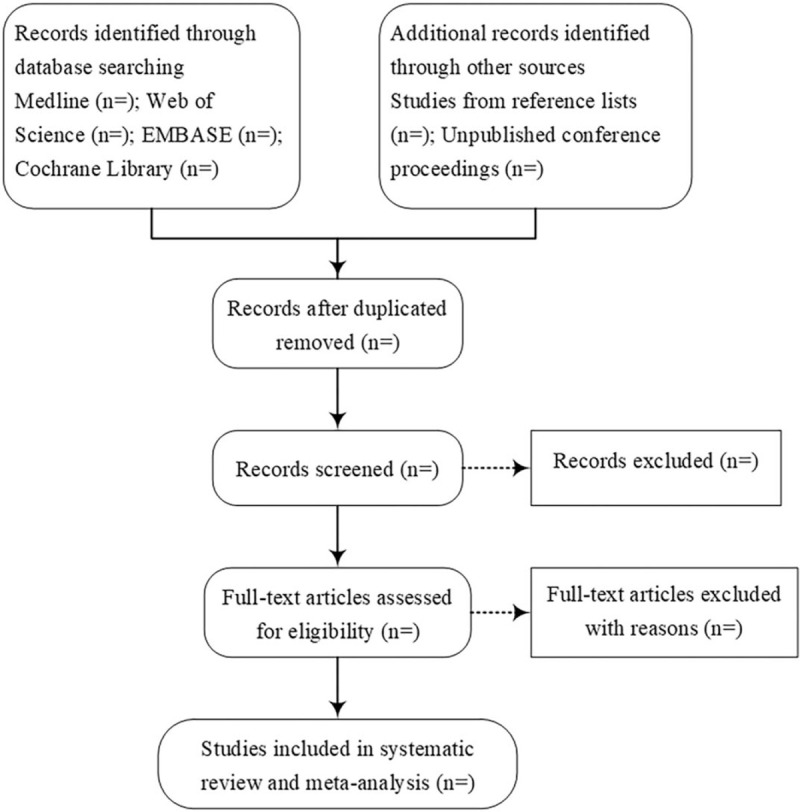
Flow diagram of study selection process.

All reviewers will follow a uniform, rigorous screening process initially negotiated and developed. Two reviewers (SC and JieZ) will screen each title and abstract to identify eligible trials according to the inclusion/exclusion criteria independently. Any disagreement will be resolved through discussion and consensus. The third reviewer (JunZ) will screen the full text if necessary when the previous 2 reviewers cannot make a clear decision. The excluded studies will be listed with reasons for their exclusion. The PRISMA flow chart will be used to illustrate the entire screening process (Fig. 1).

### 2.4 Data extraction and management

After study selection, data will be extracted and recorded in a data extraction sheet by 2 independent reviewers (SC and JieZ) using a standard form. The following data will be extracted:

(1) General information: the first author, year of publication, statistical approaches, publication source, country, funding supports.
(2) Participants: sex, age, the mean age of participants, handedness, ethnicity, education, illness duration.
(3) Study characteristics: design, sample size, diagnostic criteria, imaging modalities (fMRI, PET, SPECT), method of analysis, clinical outcome measures.
(4) Results: the altered brain regions (described with peak MNI/Talairach coordinates, cluster size, statistical threshold, correction method), the correlations of imaging data and clinical data.

Extracted data will be checked by the third reviewer (RS). Authors of included studies will be contacted if data are not available online and/or if there is a question regarding the data presented in the article. Covidence (https://www.covidence.org), an online 14 systematic review management system recommend by the Cochrane Library, will be used to manage literature.

### 2.5 Quality assessment

According to previous neuroimaging reviews,^[27,28]^ an 11-point checklist (Table 1) will be used by 2 independent reviewers (SC and YQ) to assess the quality of the included studies. The 11-point checklist concentrates on both clinical and neuroimaging methodology of each study and mainly assesses the quality from 3 aspects: subjects, methods for image acquisition and analysis, and results and conclusions. Each item will be matched with the study and then assigned a score of 1 (fully satisfied), 0.5 (partially satisfied), or 0 (otherwise). Any discrepancy will be resolved by a third reviewer (SRR). The scores will be added for the final assessment.

**Table 1:** Quality assessment checklist.

Category 1: Subjects	Score
1. Patients were evaluated prospectively, specific diagnostic criteria were applied, and demographic data were reported	
2. Healthy comparison subjects were evaluated prospectively; psychiatric and medical illnesses were excluded	
3. Important variables (e.g., age, sex, illness duration, onset time, medication status, comorbidity, severity of illness) were checked, either by stratification or statistically	
4. Sample size per group >10	
Category 2: Methods for image acquisition and analysis	
5. Magnet strength ≥1.5 T	
6. For resting-state functional MRI studies, whole brain analysis was automated with no a priori regional selection	
7. Coordinates reported in a standard space	
8. Processing of the imaging technique was described clearly enough to be reproduced	
9. Measurements were described clearly enough to be reproduced	
Category 3: Results and conclusions	
10. Statistical parameters were provided for significant and important non-significant differences	
11. Conclusions were consistent with the results obtained, and the limitations were discussed	
Total/11	

### 2.6 Data synthesis

The collected data will be presented in a table, and a qualitative review will be conducted to synthesize the cerebral functional abnormalities and the correlations between these significantly changed brain regions and the clinical variables in patients with FD.

In addition, AES-SDM (http://www.sdmproject.com/software)^[24,25,29]^ will be used to quantitatively synthesize the brain functional alterations between FD patient and HS. The units of each data from different trials will be converted to the international system of units before statistical analysis. *t*- or *P*-values for significant clusters will be converted to *z*-statistics using the SDM online converter (www.sdmproject.com/utilities/?show=Statistics). Coordinates in different stereotactic spaces will be automatically converted by SDM software provided by Radua J et,al, (London,UK).

These maps will include both activations (patients with FD patients >HS) and deactivations (patients with FD <HS) in order to correctly analyze those regions with higher between-study heterogeneity.^[30]^ In each included study, the extracted peak information (co-ordinates, significant level, and direction of change) will be combined to recreate an effect-size map and a variance map by means of a Gaussian kernel that assigns higher effect sizes to the voxels closer to the peaks; in the assignment, a relatively full-width at half-maximum (FWHM) will be set at 20 mm to control for false-positive results.^[24]^ Next, study maps will be voxel-wise calculated to acquire the random-effects mean, which takes study sample size, intra-study variability, and between-study heterogeneity into account. The mean map will be weighted by the square root of the sample size of each study; therefore, studies with larger sample sizes will contribute more to the mean map. After calculating meta-analytic means, thresholds will be applied using default settings (voxel threshold *P* < .005, peak height threshold Z > 1.00, and cluster size threshold >10 voxels).

Finally, the meta-analytic effect-size map will be statistically assessed by comparing to a null distribution created with a permutation algorithm.^[29]^ We will create funnel plots to check whether findings might have been driven by a few studies or those smaller in size. Gross abnormalities and publication bias will be formally assessed with the Egger test^[24,30]^ if possible.

We will conduct sensitivity analyses to explore the impacts of risk of trial bias on important results. Leave-one-out jackknife analysis will be used, which performs the meta-analysis again while iteratively excluding each of the papers in turn, to test the robustness of the original results.^[25]^ If there is significant heterogeneity and the available trials are sufficient, subgroup and meta-regression analysis will be performed based on the demographic data (sex, mean age, mean durations), different scan approaches (MRI, PET, and SPECT), clinical variables (NDI scores, SAS scores, and SDS scores etc), and the quality of included studies.

## 3 Results reporting and presentation

Result reporting and presentation will follow the Meta-analysis of Observational Studies in Epidemiology guidelines for reporting.^[31]^ The selection process will be summarized in a flowchart. Quantitative data will be presented in the evidence tables of individual studies and in the appropriate summary tables and forest maps. The quality scores and risk of bias for each eligible study will be reported accordingly.

## 4 Conclusion

This study summarizes the protocol for a systematic review and meta-analysis of functional neuroimaging studies conducted in adult patients with FD. This review and meta-analysis aim to qualitatively and quantitatively assess and synthesize functional cerebral abnormalities consistently found in FD. This may assist in mapping functional brain abnormalities to characterize imaging-based neural markers of FD.

The purpose of this protocol is to assess and synthesize the evidence of the impact of FD on brain function qualitatively and quantitatively. This review will be the first to synthesize neuroimaging evidence in a meta-analysis and the first to assess the quality of the included neuroimaging studies. This review and meta-analysis aim to clarify the functional anomalies underpinning this condition and improve our knowledge of the pathogenesis of FD.

### 4.1 Study status

This study has been registered on the PROSPERO on July 2, 2019 (Registration Number: CRD42019134983). Under the guidance of the PRISMA, this study has begun, and the search strategy has been established. Data extraction is planned to be finished on November 1, 2020 and the overall review will be completed on March 30, 2021.

## Author contributions

**Supervision:** Jun Zhou, Guixing Xu.

**Writing – original draft:** Ruirui Sun, Jie Zhou, Yuzhu Qu.

**Writing – review & editing:** Shirui Cheng.
